# Update on Programmed Death-1 and Programmed Death-Ligand 1 Inhibition in the Treatment of Advanced or Metastatic Non-Small Cell Lung Cancer

**DOI:** 10.3389/fonc.2017.00067

**Published:** 2017-04-06

**Authors:** Marco A. J. Iafolla, Rosalyn A. Juergens

**Affiliations:** ^1^Department of Oncology, Juravinski Cancer Centre, McMaster University, Hamilton, ON, Canada

**Keywords:** non-small-cell lung cancer, immuno-oncology, programmed death-1, programmed death-ligand 1, CTLA-4

## Abstract

**Purpose:**

Non-small-cell lung cancer (NSCLC) has a large worldwide prevalence with a high mortality rate. Chemotherapy has offered modest improvements in survival over the past two decades. Immune checkpoint modulation with programmed death-1 (PD-1) or programmed death-ligand 1 (PD-L1) inhibition has shown the promise of changing the future landscape of cancer therapy. This update reviews recent advances in the treatment of NSCLC with immune checkpoint modulation.

**Methods:**

Publications and proceedings were identified from searching PubMed and proceedings from the annual meetings of the American Society of Clinical Oncology, European Society for Medical Oncology, and European Lung Cancer Conference.

**Results:**

Atezolizumab, nivolumab, and pembrolizumab increase overall survival in second-line treatment of Stage III/IV squamous and non-squamous NSCLC when compared to docetaxel. Pembrolizumab increases progression-free survival in the first-line treatment of Stage IV NSCLC with 50% PD-L1 expression when compared to platinum-based chemotherapy. Combination therapy with chemotherapy and cytotoxic T-lymphocyte-associated protein 4 (CTLA-4) inhibitors has shown promise in early trials.

**Conclusion:**

Immune checkpoint modulation produces durable responses and overall survival benefits with less toxicity compared to conventional chemotherapy. Future investigations are combining PD-1/L1 inhibition with chemotherapy, targeted therapy, or other immuno-oncology agents in an effort to further improve efficacy.

## Introduction

More than 100 years of research in the field of cancer immunotherapy has produced several modalities capable of producing clinical response (1). Most notably, immune checkpoint modulation has shown the promise of changing the future landscape of cancer therapy through its durable clinical responses (2–4) and safety profiles of some agents that are either milder and/or more manageable compared to traditional anti-neoplastic therapies (5). Cytotoxic T-lymphocyte-associated protein 4 (CTLA-4), the first immune checkpoint receptor to be clinically targeted, is exclusively expressed on the surface of CD4^+^ and CD8^+^ T cells in lymphatic tissue and is involved in T-cell regulation, proliferation, and tolerance (6). The repertoire of immune modulation was expanded with the advent of programmed death-1 (PD-1) immune checkpoint inhibitor antibodies, which restores T-cell effector function and augments the host anti-tumor response by blocking the binding of either programmed death-ligand 1 (PD-L1) and/or PD-L2 to PD-1 receptors (7).

Following the clinical success of treating melanoma with immune checkpoint modulation (8), trialists have expanded the application of checkpoint inhibitors to multiple tumor types, including lung cancer (9). Globally, 1.8 million new diagnoses of lung cancer occurred in 2012; with a mortality rate of nearly 90%, lung cancer is the first and second cause of cancer mortality in men and women, respectively (10). Eighty-five percent of lung cancers are non-small-cell lung cancer (NSCLC), further divided into non-squamous (70%) and squamous (30%) histologic subtypes (11). Metastatic disease is present in 50% of new NSCLC diagnoses (12, 13), which harbors an untreated median overall survival (mOS) of 4.0 months (14) and a metastatic 5-year survival rate ranging from 2 to 9% (15). Although mortality has improved with the use of targeted drugs for driver mutations (16–20), few patients harbor these mutations and resistance to targeted treatment frequently occurs (21). Currently, NSCLC has numerous checkpoint inhibitors being evaluated for clinical efficacy (22). The possible treatment of NSCLC is being further enriched through the addition of other immune modulation targets and combination therapy. At present, the Food and Drug Administration has approved three immuno-oncology agents for the treatment of NSCLC: atezolizumab, nivolumab, and pembrolizumab in the relapsed, refractory setting as well as pembrolizumab for the first-line treatment of metastatic NSCLC with a tumor proportion score (TPS) ≥50%. This update will offer guidance into the current application and pending developments for treatment NSCLC with immune modulating pharmacotherapy.

## Methods

Current studies investigating the use of immune checkpoint modulation in NSCLC were reviewed by searching PubMed (January 1, 2015 to December 30, 2016) using the following search terms: non-small-cell lung cancer and immune checkpoint modulation (or aliases). Any proceedings from the American Society of Clini-cal Oncology (ASCO) (2015–2016), European Society for Medical Oncology (ESMO) (2015–2016), and European Lung Cancer Conference (ELCC) (2015–2016) annual meetings involving both NSCLC and immune checkpoint modulation were reviewed. Table 1 summarizes the search results and each trial’s pertinent characteristics.

**Table 1:** **Summary of advanced or metastatic non-small-cell lung cancer immuno-oncology trials**.

Type of treatment		Trial name	Trial phase	Stage	Histology	Programmed death-ligand 1 (PD-L1) tumor expression level	Study arm (*n*)	Comparative arm (*n*)	Primary endpoint (study versus comparator)^a^	Secondary endpoint(s) (study versus comparator, when appropriate)^a^^,^^b^	TRAEs (study versus comparator)^a^	Death from study drug (patients)^a^	Reference
Monotherapy anti-PD-1/L1	Third line	CheckMate 063	2	IIIb/IV	Squamous	≥1, ≥5, ≥10, and ≥5% for analysis	Nivolumab 3 mg/kg every 2 weeks (*n* = 117)	N/A	ORR: 14.5% (95% CI 8.7–22.2%)	mOS: 8.2 months (95% CI 6.1–10.9)	Grade 3–4: 17%	2	(23)
										median progression-free survival (mPFS): 1.9 months (95% CI 1.8–3.2)			
	Second line	CheckMate 017	3	IIIb/IV	Squamous	≥1, ≥5, and ≥10%	Nivolumab 3 mg/kg every 2 weeks (*n* = 135)	Docetaxel 75 mg/m^2^ every 3 weeks (*n* = 137)	mOS: 9.2 versus 6.0 months [hazard ratio (HR) 0.59; 95% CI 0.44–0.79; *p* < 0.001]	mPFS: 3.5 versus 2.8 months (HR 0.62; 95% CI 0.47–0.81; *p* < 0.001)	Grade 3–4: 8 versus 56%	0	(26)
										ORR: 20 versus 9% (*p* = 0.008)			
		CheckMate 057	3	IIIb/IV	Non-squamous	≥1, ≥5, and ≥10%	Nivolumab 3 mg/kg every 2 weeks (*n* = 292)	Docetaxel 75 mg/m2 every 3 weeks (*n* = 290)	mOS: 12.2 versus 9.4 months (HR 0.73; 95% CI 0.59–0.89; *p* = 0.002)	mPFS: 2.3 versus 4.2 months (HR 0.92; 95% CI 0.77–1.11; *p* = 0.39)	Grade 3–4: 10 versus 54%	0	(27)
										ORR: 19 versus 12% (*p* = 0.02)			
		Keynote 010	2 and 3	IIIb/IV	Squamous and non-squamous	≥1 and ≥50%	Pembrolizumab 2 mg/kg every 3 weeks (*n* = 345)	Docetaxel 75 mg/m2 every 3 weeks (*n* = 343)	mOS (PD-L1 expression ≥1%; pembro 2 mg/kg): 10.4 versus 8.5 months (HR 0.71; 95% CI 0.58–0.88; *p* = 0.0008)	ORR (PD-L1 expression ≥1%; pembro 2 mg/kg): 18 versus 9% (*p* = 0.0005)	Grade 3–5: 13% (pembro 2 mg/kg) and 16% (pembro 10 mg/kg) versus 35%	3 (pembro 2 mg/kg) and 3 (pembro 10 mg/kg)	(29)
							Pembrolizumab 10 mg/kg every 3 weeks (*n* = 346)		mOS (PD-L1 expression ≥1%; pembro 10 mg/kg): 12.7 versus 8.2 months (HR 0.61; 0.49–0.75; *p* < 0.0001)	ORR (PD-L1 expression ≥1%; pembro 10 mg/kg): 18 versus 9% (*p* = 0.0002)			
									mPFS (PD-L1 expression ≥1%; pembro 2 mg/kg): 3.9 versus 4.0 months (HR 0.88; 95% CI 0.74–1.05; *p* = 0.07)	ORR (PD-L1 expression ≥50%; pembro 2 mg/kg): 30 versus 8% (*p* < 0.0001)			
									mPFS (PD-L1 expression ≥1%; pembro 10 mg/kg): 4.0 versus 4.0 months (HR 0.79; 95% CI 0.66–0.94; *p* = 0.004)	ORR (PD-L1 expression ≥ 50%; pembro 10 mg/kg): 29% vs 8% (*p* < 0.0001)			
									mOS (PD-L1 expression ≥50%; pembro 2 mg/kg): 14.9 versus 8.2 months (HR 0.54; 95% CI 0.38–0.77; *p* = 0.0002)				
									mOS (PD-L1 expression ≥50%; pembro 10 mg/kg): 17.3 versus 8.2 months (HR 0.50; 95% CI 0.36–0.70; *p* < 0.0001)				
									mPFS (PD-L1 expression ≥50%; pembro 2 mg/kg): 5.0 versus 4.1 months (HR 0.59; 95% CI 0.44–0.78; *p* = 0.0001)				
									mPFS (PD-L1 expression ≥50%; pembro 10 mg/kg): 5.2 versus 4.1 months (HR 0.59; 95% CI 0.45–0.78; *p* < 0.0001)				
		OAK	3	IIIb/IV	Squamous and non-squamous	TC/IC 0 = <1% TC/IC; TC/IC 1 = ≥1% TC/IC; TC/IC 2 = ≥5% TC/IC; TC/IC 3 = ≥50% TC/≥10% IC	Atezolizumab 1,200 mg (*n* = 425) every 3 weeks	Docetaxel 75 mg/m^2^ every 3 weeks (*n* = 425)	mOS: 13.8 versus 9.6 months (HR 0.73; 95% CI 0.62–0.87; *p* = 0.0003)	mPFS: 4.0 versus 2.8 months (HR 0.95; 95% CI 0.82–1.10; *p* = 0.493)	Grade 3–4: 15 versus 43%	0	(31)
									mOS (PD-L1 expression ≥1%): 15.7 versus 10.3 months (HR 0.74; 95% CI 0.58–0.93; *p* = 0.0102)				
	First line	Keynote 024	3	IV	Squamous and non-squamous	≥50%	Pembrolizumab 200 mg every 3 weeks (*n* = 154)	Investigator’s choice of five different platinum-based chemotherapy regimens (*n* = 150)	mPFS (PD-L1 expression ≥50%): 10.3 versus 6.0 months (HR 0.50; 95% CI 0.37–0.68; *p* < 0.001)	mOS (yet to be reached). Six-month survival (PD-L1 expression ≥5%): 80.2% versus 72.4% (HR 0.60; 95% CI 0.41–0.89; *p* = 0.005)	Grade 3–5: 26.6 versus 53.3%	0	(32)
										ORR (PD-L1 expression ≥5%): 44.8 versus 42% (*p* value not reported)			
		CheckMate 026	3	IV or recurrent	Squamous and non-squamous	≥1 and ≥5%	Nivolumab 3 mg/kg every 2 weeks (*n* = 271)	Investigator’s choice of platinum-based doublet chemotherapy (*n* = 270)	mPFS (PD-L1 expression ≥5%): 4.2 versus 5.9 months (HR 1.15, 95% CI 0.91–1.45; *p* = 0.25)	mPFS: not reported	Grade 3–4: 18 versus 51%	Not stated	(33)
										mOS: 14.4 versus 13.2 months (HR = 1.02; 95% CI: 0.80–1.30) (*p* value not stated)			
										ORR: not reported			
Combination chemotherapy and anti-PD-1 therapy		CheckMate 012	1	IIIb/IV	Squamous and non-squamous	<1 and ≥1%	Squamous: nivolumab 10 mg/kg every 3 weeks plus gemcitabine-cisplatin (*n* = 12)	N/A	Tolerability: 21% discontinuation from TRAE; TRAE (grade 3–4): 45%	ORR (nivo 10 mg/kg plus gem-cis): 33%	Grade 3–4: 45%	0	(35)
							Non-squamous: nivolumab 10 mg/kg every 3 weeks plus pemetrexed-cisplatin (*n* = 15)			ORR (nivo 10 mg/kg plus pem-cis): 47%			
							All histologies: nivolumab 5 or 10 mg/kg every 3 weeks plus paclitaxel-carboplatin (*n* = 14 and *n* = 15, respectively)			ORR (nivo 10 mg/kg plus pacli-carbo): 47%			
										ORR (nivo 5 mg/kg plus pacli-carbo): 43%			
										progression-free survival (PFS) (24 weeks; nivo 10 mg/kg plus gem-cis): 51%			
										PFS (24 weeks; nivo 10 mg/kg plus pem-cis): 71%			
										PFS (24 weeks; nivo 10 mg/kg plus pacli-carbo): 38%			
										PFS (24 weeks; nivo 5 mg/kg plus pacli-carbo): 51%			
		Keynote 021	1 and 2	IIIb/IV	Non-squamous	<1 and ≥1%	Pembrolizumab 200 mg every 3 weeks plus pemetrexed-carboplatin (*n* = 60)	Pemetrexed-carboplatin (*n* = 63)	ORR: 55 versus 29% (95% CI 9–42%; *p* = 0.0016)	mPFS: 13.0 versus 8.9 months (HR 0.53; 95% CI 0.31–0.91; *p* = 0.010)	Grade 3–5: 39 versus 26%	1	(36)
										OS (12 months): 75 versus 72%			
										ORR (<1versus ≥1% in pembro arm): 57 versus 54%			
										ORR (PD-L1 1–49% in pembro arm): 26%			
										ORR (PD-L1 ≥50% in pembro arm): 80%			
Combination anti-PD-1/L1 therapy and anti-CTLA-4 therapy		Keynote 021	1 and 2	IIIb/IV	Non-squamous	<1 and ≥1%	Maximum dose: Pembrolizumab 10 mg/kg every 3 weeks plus ipilimumab 1 or 3 mg/kg every 3 weeks (only four cycles)	N/A	Dose-limiting toxicities: none	None defined	All grade: 10 patients (66.7%)	0	(37)
							Final dose selected: pembrolizumab 2 mg/kg and ipilimumab 1 mg/kg		TRAE (all grades): 10 patients (66.7%)				
		CheckMate 012	1	IIIb/IV	Squamous and non-squamous	<1 and ≥1%	Nivolumab 1 mg/kg every 2 weeks plus ipilimumab 1 mg/kg every 6 weeks (data not reported in publication)	N/A	TRAE (grade 3–4; ipi every 6 weeks): 33%	ORR (ipi every 6 weeks): 38% (95% CI 23–55)	TRAE (grade 3–4; ipi every 6 weeks): 33%	0	(39)
							Nivolumab 3 mg/kg every 2 weeks plus ipilimumab 1 mg/kg every 12 weeks (*n* = 38)		TRAE (grade 3–4; ipi every 12 weeks): 37%	ORR (ipi every 12 weeks): 47% (95% CI 31–64)	TRAE (grade 3–4; ipi every 12 weeks): 37%		
							Nivolumab 3 mg/kg every 2 weeks plus ipilimumab 1 mg/kg every 6 weeks (*n* = 39)			PFS (24 weeks; ipi every 6 weeks): 65% (95% CI 42–81)			
										PFS (24 weeks; ipi every 12 weeks): 80% (95% CI 55–92)			
		D4190C00006	1b	III/IV	Squamous and non-squamous	Unknown, 0, <25, and ≥25%	Maximum dose: durvalumab 20 mg/kg with tremelimumab 3 mg/kg	N/A	Serious adverse event (“serious” not formally defined): 37%	ORR (durvalumab 10–20 mg/kg every 2 weeks or 4 weeks plus tremelimumab 1 mg/kg): 23% (95% CI 9–44)	Serious adverse event (“serious” not formally defined): 37%	3	(40)
							Final dose selected: durvalumab 10 mg/kg and tremelimumab 1 mg/kg, both every 4 weeks (*n* = 102)						

*mOS, median overall survival; mPFS, median progression-free survival; n, number of patients; ORR, overall response rate; TRAE, treatment-related adverse event*.

*^a^Results are for all studied patients, unless otherwise stated*.

^b^Only selected secondary endpoints reported in Table 1

## Results

### Third Line

CheckMate 063 is a Phase 2, open-label, global, multicenter, single-arm trial investigating the use of nivolumab, a fully human immunoglobulin G4 (IgG4) monoclonal antibody that selectively inhibits the PD-1 receptor, dosed 3 mg/kg every 2 weeks (*n* = 117) in patients with either Stage IIIb/IV squamous NSCLC who have received prior platinum-doublet and one additional systemic treatment. Treatment with nivolumab continued until progressive disease (PD) or an unacceptable treatment-related adverse event (TRAE), although treatment beyond PD was permitted as per protocol. The primary endpoint was overall response rate (ORR) by independent radiology review (per RECIST v1.1). The ORR was 14.5% (95% CI 9–22). mOS was 8.2 months (95% CI 6.1–10.9), with 12-month OS and 18-month OS rates of 39% (95% CI 30–48) and 27% (95% CI 19–35), respectively. TRAE of any Grade occurred in 75% of patients, Grade 3–4 TRAEs occurred in 17%, TRAE lead to nivolumab discontinuation in 12%, and death occurred in two patients secondary to nivolumab, although these patients had multiple comorbidities in the setting of PD (23, 24). These results are similar to those obtained from two smaller Japanese trials (25). To put this in historical perspective, a retrospective analysis looking at third-line treatment (58% received cytotoxic chemotherapy, 42% EGFR received tyrosine kinase inhibitors) in patients who had not received any immunotherapy found a 6.5-month mOS, 3.4-month median progression-free survival (mPFS), and 8% ORR (26).

### Second Line

CheckMate 017 is a Phase 3, global, multicenter, open-label, 1:1 randomized trial comparing nivolumab 3 mg/kg every 2 weeks (*n* = 135) or docetaxel 75 mg/m^2^ every 3 weeks (*n* = 137) in patients with Stage IIIb/IV squamous NSCLC histology that recurred or progressed following prior platinum-doublet therapy. Treatment continued until PD or unacceptable toxicity, and treatment beyond PD was allowed. The primary endpoint was OS. Nivolumab had an mOS of 9.2 months (95% CI 7.33–12.62) versus docetaxel with 6.0 months (95% CI 5.29–7.39). The risk of death was 41% lower with nivolumab versus docetaxel [hazard ratio (HR) 0.59, 95% CI 0.44–0.79; *p* < 0.001]. The ORR with nivolumab was 20 versus 9% with docetaxel. PD-L1 expression stratified to 1, 5, and 10% was not predictive of benefit. Nivolumab had less TRAEs compared to docetaxel: TRAEs of any grade were reported in 59 versus 87% of patients, respectively, and Grades 3–4 TRAEs were reported in 8 versus 56% of patients, respectively. Study discontinuation due to a TRAE was reported for 5% of nivolumab versus 10% of docetaxel patients. No treatment-related deaths occurred with nivolumab and two deaths occurred in patients receiving docetaxel that were determined to be treatment-related (27). This efficacy is similar to the single-arm CheckMate 063 results (23). OS was updated at the ASCO 2016 Annual Meeting: the 1- and 2-year OS for nivolumab was 42 and 23%, respectively, in comparison to 24 and 8% for docetaxel (28).

CheckMate 057 is a Phase 3, global, multicenter, open-label, 1:1 randomized trial comparing nivolumab 3 mg/kg every 2 weeks (*n* = 292) to docetaxel 75 mg/m^2^ every 3 weeks (*n* = 290) in patients with Stage III/IV non-squamous NSCLC that recurred or progressed on platinum-doublet chemotherapy. Treatment continued until PD or discontinuation due to toxicity; treatment beyond PD was permitted per protocol. This study met its primary endpoint of OS, with nivolumab mOS 12.2 months versus docetaxel 9.4 months, yielding a 27% reduction in risk of death (HR 0.73; *p* = 0.002) and improved ORR (19 versus 12%; *p* = 0.02). Using pre-defined PD-L1 expression levels of ≥1, ≥5, and ≥10% from archival tumors, nivolumab showed improved efficacy across all endpoints. PD-L1 expression predicted the benefit of nivolumab, even at the lowest expression level of 1%. mOS approximately doubled with nivolumab versus docetaxel across PD-L1 expression levels; conversely, survival was equivocal with negative PD-L1 expression. Grades 3–4 TRAEs occurred in 10 and 54% of nivolumab and docetaxel patients, respectively. There were no nivolumab-related deaths, whereas docetaxel led to one death. Subsequent systemic therapy was given to 42.1 and 49.7% of nivolumab and docetaxel patients, respectively (29). Investiga-tors at the ASCO 2016 Annual Meeting presented an update to the 1- and 2-year OS for nivolumab noting 51 and 29%, respectively, in comparison to 39 and 16% for docetaxel (28).

Keynote 010 is Phase 2/3, global, multicenter, open-label, randomized, controlled study to evaluate the safety and efficacy of pembrolizumab, a humanized monoclonal IgG4 antibody that selectively inhibits the PD-1 receptor. Patients with NSCLC and tumor cell PD-L1 expression ≥1% who progressed after platinum-doublet chemotherapy were randomized 1:1:1 to receive pembrolizumab 2 mg/kg (*n* = 345), pembrolizumab 10 mg/kg (*n* = 346), or docetaxel 75 mg/m^2^ (*n* = 343) every 3 weeks; no crossover was allowed. Treatment was continued for 24 months, or until PD or discontinuation due to toxicity; treatment beyond PD was allowed. The primary endpoints were OS and progression-free survival (PFS) (by independent radiology review as per RECIST v1.1) in both all patients and those with PD-L1 expression ≥50% of tumor cells (TCs) from either archival or new biopsies. Compared to docetaxel, risk of death was decreased with both pembrolizumab 2 mg/kg (HR 0.71, 95% CI 0.58–0.88; *p* = 0.0008) and pembrolizumab 10 mg/kg (HR 0.61, 0.49–0.75; *p* < 0.0001). Patients with PD-L1 expression ≥50% had better mOS with pembrolizumab 2 mg/kg (14.9 versus 8.2 months; HR 0.54, 95% CI 0.38–0.77; *p* = 0.0002) and pembrolizumab 10 mg/kg (17.3 versus 8.2 months; HR 0.50, 95% CI 0.36–0.70; *p* < 0.0001) versus docetaxel. Grades 3–5 TRAEs were less common with pembrolizumab versus docetaxel: 13, 16, and 35% for those treated with pembrolizumab 2 mg/kg, pembrolizumab 10 mg/kg, and docetaxel, respectively (30). Investigators at the ASCO 2016 Annual Meeting presented an update on the patients with 1–49% PD-L1 expression: OS was longer for both pembrolizumab 2 mg/kg (9.4 months) versus docetaxel (8.6 months) (HR 0.79, 95% CI 0.61–1.04) and pembrolizumab 10 mg/kg (10.8 months) versus docetaxel (8.6 months) (HR 0.71, 0.53–0.94) (31).

The OAK study is a Phase 3, global, multicenter, open-label, randomized, controlled study to evaluate the efficacy and safety of atezolizumab, a humanized IgG1-kappa monoclonal antibody that binds PD-L1 and inhibits PD-L1/PD-1 and PD-L1/B7.1 interactions (32). Patients with Stage IIIb/IV or recurrent non-squamous NSCLC following failure of platinum-based treatment were randomized 1:1 to receive either fixed dose atezolizumab 1,200 mg (*n* = 425) or docetaxel 75 mg/m^2^ (*n* = 425) every 3 weeks; no crossover was allowed. Treatment continued until disease progression or unacceptable toxicity occurred; treatment beyond PD was allowed. The study had co-primary endpoints of OS in the full study population, in addition to OS in patients with PD-L1 expression ≥1% of TCs or tumor-infiltrating immune cells (IC) by the Ventana SP142 assay. mOS was significantly longer for atezolizumab versus docetaxel (13.8 versus 9.6 months, stratified HR 0.73, 95% CI 0.62–0.87; *p* = 0.0003), and had 12- and 18-month OS of 55 versus 41% and 40 versus 27%, respectively. OS was significant regardless of presence of PD-L1 expression: 55% of patients had PD-L1 expression ≥1% and had OS of 15.7 months versus docetaxel 10.3 months (stratified HR 0.74, 95% CI 0.58–0.93; *p* = 0.0102); 45% of patients had no TC or IC with PD-L1 expression with a respective OS of 12.6 months versus docetaxel 8.9 months (HR 0.75, 95% CI 0.59–0.96; *p* = 0.0215); and 16% of patients had high TC (≥50%) or IC (≥10%) PD-L1 expression and had OS of 20.5 months versus docetaxel 8.9 months (HR 0.41, 95% CI 0.27–0.64; *p* < 0.0001). Further, OS was also significant regardless of NSCLC histology: non-squamous NSCLC had OS of 15.6 months versus docetaxel 11.2 months (HR 0.73, 95% CI 0.60–0.89; *p* = 0.0015); squamous NSCLC had OS of 8.9 months versus docetaxel 7.7 months (HR 0.73, 95% CI 0.54–0.98; *p* = 0.0383). Five percent of the atezolizumab group went on to receive subsequent immunotherapy versus 17% in the docetaxel group. Atezolizumab was well tolerated: only 15% of patients treated with atezolizumab had Grades 3–4 adverse events compared to 43% of the patients treated with docetaxel (33).

### First Line

Keynote 024 is Phase 3, global, multicenter, open-label, 1:1 ran-domized trial comparing fixed dose pembrolizumab 200 mg every 3 weeks (*n* = 154) to the investigator’s choice of five different platinum-based chemotherapy regimens (*n* = 150) in patients with both squamous and non-squamous Stage IV NSCLC who have not received prior systemic therapy for their metastatic disease and have PD-L1 expression on ≥50% of TCs. Treatment with pembrolizumab and platinum-based chemotherapy continued for a total of 35 cycles (~2 years) and 4–6 cycles, respectively, or until the patient had radiologic disease progression or unacceptable toxicity. Pemetrexed maintenance was allowed for patients with non-squamous histology. Crossover from chemotherapy to pembrolizumab was allowed if PD occurred. The primary end point was PFS (by independent radiology review as per RECIST v1.1). mPFS was longer for pembrolizumab versus chemotherapy (10.3 versus 6.0 months) and disease progression or death was significantly better for pembrolizumab (HR 0.50, 95% CI 0.37–0.68; *p* < 0.001). mOS has yet to be reached; however, the 6-month OS for pembrolizumab versus chemotherapy was 80.2 and 72.4%, respectively (HR 0.60, 95% CI 0.41–0.89; *p* = 0.005). Pembrolizumab had fewer TRAEs of any grade compared to chemotherapy (73.4 versus 90.0%), less grade 3–5 TRAEs (26.6 versus 53.3%), and although had higher rates of immune-TRAEs (29.2 versus 4.7%) most were grade 1–2 severities and did not lead to any deaths. The second interim analysis by the data and safety monitoring committee determined that the benefit of pembrolizumab was large enough to warrant stopping the trial and offer the chemotherapy group pembrolizumab (34).

CheckMate 026 is a Phase 3, global, multicenter, open-label, 1:1 randomized controlled trial comparing nivolumab 3 mg/kg every 2 weeks (*n* = 271) to the investigator’s choice of platinum-based doublet chemotherapy (*n* = 270) in patients with Stage IV or recurrent squamous and non-squamous NSCLC who have not received previous systemic therapy for their disease and have PD-L1 expression on ≥1% of TCs. Nivolumab continued until disease progression or unacceptable toxicity. Treatment beyond progression was allowed. Platinum-doublet chemotherapy was given for up to six cycles and pemetrexed maintenance was allowed for non-squamous patients. Crossover from chemotherapy to nivolumab was allowed if PD occurred. The primary end point was PFS (by independent radiology review as per RECIST v1.1) in patients with PD-L1 expression ≥5%. Nivolumab did not improve mPFS compared to platinum-doublet among patients with PD-L1 expression ≥5%: 4.2 and 5.9 months, respectively (HR 1.15, 95% CI 0.91–1.45; *p* = 0.25). Surprisingly, in contrast to the Keynote 024 results, the PFS was not superior in the ≥50% PD-L1 cohort (PFS HR 1.07) in the subgroup analysis. The OS for the nivolumab and chemotherapy groups were similar with an mOS of 14.4 and 13.2 months, respectively (HR = 1.02, 95% CI 0.80–1.30). Nivolumab had less TRAEs of any grade and grade 3–4 (71 and 18%), respectively, when compared to platinum-doublet (92 and 51%) (35). Further knowledge on the use of first-line nivolumab will be forthcoming from the ongoing CheckMate 227 trial where the 1% PD-L1 positive arm includes patients randomized to combined immunotherapy with nivolumab and ipilimumab versus nivolumab alone versus chemotherapy in the first-line setting.

### Combinations of Immunotherapy and Chemotherapy

CheckMate 012 is a multi-cohort phase I clinical trial evaluating nivolumab as a single agent or in combination with chemotherapy, targeted therapy, or ipilimumab (a recombinant human IgG1 immunoglobulin that inhibits the CTLA-4 receptor). The data have been published from the platinum-doublet combi-nations (36). Three chemotherapy backbones were evaluated in 56 patients: pemetrexed/cisplatin, gemcitabine/cisplatin, and paclitaxel/carboplatin. All three backbones were paired with nivolumab 10 mg/kg. An additional cohort of paclitaxel/carboplatin was accrued that was combined with nivolumab 5 mg/kg. Maintenance chemotherapy was not allowed but patients continued on maintenance of nivolumab until progression. The primary objective, ORR, was 47, 33, and 47% for the pemetrexed, gemcitabine and paclitaxel platinum combinations with the 10 mg/kg nivolumab dose, respectively; ORR for paclitaxel/carboplatin combination with 5 mg/kg nivolumab was 43%. Responses were seen irrespective of presence or absence of PD-L1 expression on the tumor. Two-year OS rates were 33, 25, and 27%, for the pemetrexed, gemcitabine and paclitaxel platinum combinations with the 10 mg/kg nivolumab dose, respectively. The 2-year OS rate for the paclitaxel/carboplatin combination with 5 mg/kg nivolumab was 62%. These objective response and 2-year survival rates for the nivolumab combinations were numerically increased over what we would have expected historically from platinum doublet. No dose-limiting toxicities occurred during the first two cycles of treatment. Forty-five percent of patients reported Grade 3–4 TRAEs; 21% of patients discontinued all study therapy as a result of TRAEs.

Keynote 021 is a multi-cohort Phase 1/2 randomized trial investigating the safety, tolerability, and efficacy of pembrolizumab in combination with platinum doublets, targeted therapy, and ipilimumab. The data were recently published from the randomized phase 2 cohort G which compared pemetrexed and carboplatin followed by pemetrexed maintenance with or without a maximum of 2 years of pembrolizumab in 123 patients. Patients have Stage IIIb/IV non-squamous NSCLC and were stratified according to their PD-L1 TPS <1 versus ≥1%. Crossover from the chemotherapy group to the pembrolizumab group was permitted in the event of PD. The primary endpoint was ORR (by independent radiology review as per RECIST v1.1). Pembrolizumab combined with chemotherapy has a superior ORR versus chemotherapy alone (55 versus 29%, 95% CI 9–42%; *p* = 0.0016). Subgroup analysis of PD-L1 stratification <1% versus ≥1% showed similar ORR for the pembrolizumab group (57 versus 54%, respectively) while the chemotherapy alone group showed a difference in ORR (13 versus 38%, respectively). Further stratification of PD-L1 to 1–49% and ≥50% had an ORR of 26 and 80%, respectively, for the pembrolizumab with chemotherapy group, versus 39 and 35%, respectively, for the chemotherapy alone group. Pembrolizumab with chemotherapy was able to achieve a superior mPFS versus chemotherapy alone (13.0 versus 8.9 months, HR 0.53, 95% CI 0.31–0.91; *p* = 0.0102). mOS has not yet been met, and the 12-month OS has been 75% for those with pembrolizumab and chem-otherapy versus 72% for chemotherapy alone. Grade 3–5 TRAEs were similar between groups (39% in the pembrolizumab plus chemotherapy group versus 26% in the chemotherapy alone group), with similar treatment discontinuation rates (10% for the pembrolizumab arm compared to 13% for the chemotherapy only arm) and treatment-related deaths (one death in the pembrolizumab group secondary to sepsis, and two deaths in the chem-otherapy alone group due to sepsis and pancytopenia) (37).

### Immunotherapy Doublets

Based on the success of dual immunotherapy combinations in melanoma, PD-1 inhibitors have been combined with the CTLA-4 inhibitor, ipilimumab. Cohort C from the Keynote 021 trial described above was presented at the ASCO Annual Meeting in 2015 (38). This cohort was a dose finding and safety study. The initial doses of pembrolizumab were 10 mg/kg and doses of 1 or 3 mg/kg of ipilimumab were planned. There were no safety signals at the 10 mg/kg dose of pembrolizumab with either dose of ipilimumab in the six patients treated, but, based on the emerging results from the CheckMate 012 trial, the final dose selected for further dose expansion was pembrolizumab 2 mg/kg and ipilimumab 1 mg/kg. Results were presented for the 15 patients treated at all dose levels. The ORR was 39% and the disease control rate was 83%. Immune TRAEs were identified in 33% of patients, half of whom had Grade 3 events (adrenal insufficiency and skin eruptions). This combination is being explored further in a randomized two cohort of Keynote 021 (cohort H).

The CheckMate 012 trial had several arms assessing the optimal dosing of nivolumab and ipilimumab in chemotherapy-naïve patients with NSCLC. Initial dose combinations with higher doses of ipilimumab (3 mg/kg) given every 3 weeks, similar to those used in melanoma, were not tolerable and had very high rates of TRAEs (49%) and treatment-related deaths in the NSCLC population (39). This prompted reassessment of dose and schedule. The results of nivolumab 3 mg/kg every 2 weeks in combination with ipilimumab 1 mg/kg every 6 or 12 weeks in 77 previously untreated metastatic NSCLC patients have been recently published (40). The ORR was 38 and 47% for the Q6 and Q12 week ipilimumab cohorts, respectively. In patients with PD-L1 expression of 1% or greater, the ORR was 57% for both cohorts. OS at 1 year in the ipilimumab Q6 week cohort was 69%. The follow-up data were too immature at the time of publication to report the OS in the Q12 week cohort. Grade 3–4 TRAEs occurred in 33 and 37% of patients in the Q6 and Q12 week cohorts, respectively. The majority of these TRAEs were auto-immune phenomena. No treatment-related deaths occurred. The results of the CheckMate 012 trial are the basis for the CheckMate 227 trial where PD-L1 positive (1%) patients are randomized to nivolumab 3 mg/kg every 2 weeks with ipilimumab 1 mg/kg every 6 weeks versus nivolumab 3 mg/kg every 2 weeks versus platinum doublet. The PD-L1 negative patients are randomized to nivolumab with ipilimumab at the dose and schedule above versus nivolumab with platinum-doublet chemotherapy versus standard of care chemotherapy.

The phase Ib experience with the combination of durvalumab, an IgG1 antagonist antibody that binds PD-L1 and inhibits its function, with tremelimumab, a fully human IgG2 isotype that inhibits the CTLA-4 receptor, in NSCLC has recently been published (41). In this dose-finding study, 102 patients were enrolled; 94% of the patients had prior systemic therapy. The final toler-able dose was established as durvalumab 10 mg/kg and tremelimumab 1 mg/kg both given every 4 weeks. Serious TRAEs occurred in 37 (36%) of 102 patients. Three treatment-related deaths occurred from suspected or confirmed autoimmunity (myasthenia gravis, pericardial effusion, and neuromuscular dis-order). In the final cohort of 26 patients treated at the tremelimumab dose of 1 mg/kg, patients with both PD-L1 high (25%) as well as PD-L1 negative (0%) tumors had ORR 22 and 40%, respectively. Further work with this combination is being done in chemotherapy refractory (ARCTIC study) (42) and chemotherapy-naïve (MYSTIC study) (43) NSCLC patients.

### PD-L1 Expression

Currently there are at least six monoclonal antibodies to assay PD-L1. The 28-8 antibody has been developed in conjunction with nivolumab. The 22C3 antibody has been developed with pembrolizumab. The 78-10 antibody has been developed with avelumab. Each of these three antibodies was developed initially on the DAKO autostainer platform. The SP142 antibody has been developed with atezolizumab. The SP263 antibody has been developed with durvalumab. Both of these antibodies were validated initially using the Ventana platform. Additional work has been published with the E1L3N antibody, which is commercially available and has been used in multiple laboratory-developed tests at numerous academic centers with both the DAKO and Ventana platforms. Work is now underway to cross compare the antibodies. Several studies have now been published including phase 1 of the BluePrint PD-L1 Assay Comparison Project (44). The results of this work consistently note that the 28-8, 22C3, and SP263 antibodies are comparable when staining tumor cells. The SP142 antibody has more variability when compared to the other three antibodies. In general, all four antibodies have greater variability when assaying ICs. Work is also underway to assess the reliability of some of the antibodies on alternative staining platforms. Recently, the Ventana platform has been shown to also be reliable for 22C3 analysis (45). While the FDA has approved many of these antibodies as either companion or complementary diagnostics, due to the high cost of these tests, globally, laboratory-developed assays for PD-L1 are likely to predominate. At this point, the authors recommend that a well-validated assay be used to determine the presence or absence of PD-L1 staining. The key to this is the requirement for rigorous validation metho-dology if a laboratory-developed assay is going to be used. This sentiment has been demonstrated in the recently presented Multi-center French Harmonization Project (46). The 22C3, 28-8, SP263, and E1L3N antibodies were generally comparable. This study did show significant variability in the detection of PD-L1-positive tumor cells when laboratory-developed tests were used. The key is a thorough initial and ongoing validation process for laboratory-developed tests.

With that background, understanding the molecular determinants of response to immunotherapies is a difficult clinical challenge. Presently, PD-L1 expression levels have shown a variable ability to predict response to checkpoint inhibition. CheckMate 017 did not show any clear predictive benefit of PD-L1 analysis at the reported 1, 5, and 10% cut points for squamous histology patients. CheckMate 057 did show significant improvement in ORR, PFS, and OS with nivolumab for non-squamous patients expressing any level of PD-L1, but there is clear escalating benefit with increasing PD-L1 expression in the published 1, 5, and 10% cut points (47). The OAK study also showed that atezolizumab, when compared to docetaxel, produced OS benefit regardless of PD-L1 expression on either TC or IC, but again, increased magnitude of benefit is seen when patients with increasing PD-L1 expression are identified (33). The Keynote 010 trial only included PD-L1 positive patients, and although it does not offer information about the PD-L1-negative patients, there was increasing benefit when the patients with low expression (1–49%) are contrasted with the patients with high expression (≥50%) (31). Consistently in patients with non-squamous tumors who have progressed on platinum doublet, there is increased chance of benefit with increased PD-L1 expression. The struggle for clinicians is that the benefit in the PD-L1 low and/or negative groups is not zero, nor is it clinically insignificant, making use of PD-L1 as a biomarker in the refractory setting a challenge.

There has been documented success when stratifying patients for PD-L1 using the 22C3 antibody at the 50% cut point. This cut point was clinically validated during the Keynote 010 phase I trial that showed both pembrolizumab at either 2 or 10 mg/kg dose significantly improved OS in patients with ≥50% PD-L1 expression, which was numerically greater than the benefits seen in the low expressing cohort (48). Keynote 024 also demonstrated dramatic benefits of pembrolizumab in comparison to platinum-doublet chemotherapy in previously untreated patients with PD-L1 expression ≥50%. This comes in contrast to what has been seen with the 28-8 antibody. The results of the CheckMate 026 trial are perplexing. If the 22C3 and 28-8 antibodies select patients similarly, as is suggested by several recent publications including the initial publication of the BluePrint PD-L1 Assay Comparison Project, one would expect the patients treated with nivolumab who had ≥50% PD-L1 expression to do better with immu-notherapy than chemotherapy, but this was not demonstrated (44, 49). The CheckMate 026 study was not designed nor powered to look at this subgroup. Confirmatory information about the benefits of PD-L1 inhibition in the chemotherapy-naïve first-line setting is needed. The Keynote 042 trial is ongoing (50). This trial is similar to the CheckMate 026 trial where patients with ≥1% PD-L1 staining are eligible to enroll. Patients are stratified by PD-L1 expression using the 50% cut point and randomized to pembrolizumab versus standard platinum-doublet chemotherapy. The high expressing group can then be used to confirm the Keynote 024 data. As mentioned earlier, the ongoing Checkmate 227 trial has a nivolumab monotherapy arm for patients with ≥1% PD-L1 expression. Again, this trial was not designed to look specifically at the 50% PD-L1-positive group but may yield a signal as to whether there is benefit of nivolumab alone in the first-line setting.

### Other Potential Biomarkers

A multivariate exploratory analysis of baseline serum cytokines levels in 222 nivolumab-treated patients in either Checkmate 017 or 063 trials was presented at the April 2016 ELCC (51). The effect of the cytokine set on OS was quantified by generating an SQ-cytoscore defined as “high” or “low” based on the median cut-off. Those with a high SQ-cytoscore (*n* = 102) achieved an mOS of 15.6 months, approximately three times longer than 5.3 months of those with a low SQ-cytoscore (*n* = 120) (HR 0.48, 95% CI 0.36–0.64; *p* < 0.0001), respectively. While clinical factors are not suitable to determine sensitivity to PD-1 inhibition and PD-L1 expression does not predict response in squamous-NSCLC, the SQ-cytoscore may serve as a predictive marker for anti-PD-1 therapy. Prospec-tive validation of these preliminary findings is required.

Due to tumor heterogeneity and the fluctuant infiltration of ICs into the tumor microenvironment (52), future biomarker investigations may look at other checkpoint molecules (53), TIIC (54), blood-based immune analyses (55), inflammatory gene signatures (56), and mutational load (57). In two independent cohorts, whole-exome sequencing of patients with NSCLC treated with pembrolizumab found an improved ORR, PFS, and durability in patients with higher tumor non-synonymous mutational load. Mutation burden was also associated with DNA repair pathway mutations, larger neoantigen burden, and molecular smoking signature; each factor was also correlated with clinical efficacy (57). Mutational burden is largely a consequence of chronic exposure to mutagens, and hence non-smoking NSCLC patients tend to harbor fewer mutations (58). Future prospective studies will need to explore the role of smoking exposure to durability of response.

### Future Work

Future trials will continue to explore the potential of combination therapy of PD-1 inhibition with chemotherapy, targeted therapy, or other immuno-oncology agents. Chemotherapy has been shown to increase PD-L1 expression on TCs (59, 60), in addition to possibly reducing the quantity and activity of suppressive ICs, inducing immunogenic cell death, activating and maturing dendritic cells, enhancing tumor antigen presentation, and increasing effector T-cell function (61). Beyond cytotoxic chemotherapy combinations, the PD-1 inhibitors are being combined with targeted therapies such as the EGFR and ALK inhibitors. As listed above, there are numerous clinical trials currently investigating the potential of combination immunotherapy in NSCLC, of which the majority are investigating the combination of PD-1/PD-L1 inhibition with CTLA-4 inhibition, but other novel checkpoint inhibitors are also entering phase I development. Studies are also investigating the role of PD-1 inhibition in the adjuvant setting in the ANVIL (nivolumab), PEARLS (pembrolizumab), IMpower010 (atezolizumab), and BR31 (durvalumab) trials as well as in locally advanced disease (PACIFIC). Beyond investigating immunotherapy in earlier stages of NSCLC, further work needs to be done to understand the mechanisms of resistance to this class of drugs. Loss-of-function mutations in the interferon-receptor-associated Janus kinase 1 (JAK1) and/or Janus kinase 2 (JAK2) genes in melanoma and mismatch repair-deficient colon cancer have been implicated in acquired resistance, or possibly even primary resistance, to PD-1 inhibition (62, 63). JAK1/2 is required for signaling through the interferon-γ receptor, a process required for TC PD-L1 expression (9). Hence inactivating JAK1/2 mutations lead to loss of PD-L1 expression and lack of response to anti-PD-1/L1 therapy. While this mechanism has yet to be studied in NSCLC, it warrants further exploration as a possible biomarker of resistance. This paper also noted changes in the folding and localization of major histocompatibility complex (MHC) 1 to the cell surface in patients who have developed resistance to PD-1/L1 checkpoint inhibition through mutations in the beta-2-microglobulin gene. The presence of MHC on the surface of tumor cells is required for T-cell cytotoxicity and lack of presence of MHC on the surface will mitigate the effect of PD-1/L1 checkpoint inhibition.

## Conclusion

Based on the data we have to date, in patients who have not received prior therapy for metastatic NSCLC, pembrolizumab could be offered as an alternative to platinum-doublet chemotherapy for those patients who express PD-L1 on ≥50% of their TCs based on the Keynote 024 trial. Confirmatory data will be forthcoming to support this single positive trial. In the refractory setting, nivolumab, pembrolizumab, and atezolizumab have all shown benefit in phase 3 clinical trials. Nivolumab is the only agent tested in a phase 3 trial with both known and unknown PD-L1 expression and demonstrated an OS benefit. Atezolizumab has shown a significant survival benefit in both the PD-L1 positive and negative patients (patients in this trial were required to have adequate tissue to document PD-L1 status). Pembrolizumab should only be given to patients who have known PD-L1 expression of at least 1%. We look forward to further phase 3 randomized data of the immunotherapy combination strategies but for now this strategy should be reserved for clinical trials.

Anti-PD1 and PD-L1 in NSCLC treatment have durable response rates of approximately 20% that produce remarkable long-term survival. Toxicity is more favorable than chemotherapy, however, unique to immune checkpoint blockade are immune TREAs, of which the Grade 3–4 occurring in 5% of patients leads to treatment discontinuation. PD-L1 expression levels show a variable response to checkpoint inhibition, and at present they are not essential to guide therapy in the patients who have failed platinum doublet. In contrast, PD-L1 expression appears to be critical in assessing the potential benefit of PD-1 inhibition in the chemotherapy-naïve patient population. Determining predictive biomarkers to response is still undergoing further investigation. The rapidly evolving future of immunotherapy will continue with future studies investigating the potential of PD-1 inhibition in combination with chemotherapy, targeted therapy, or other immuno-oncology agents. We have entered a new era of lung cancer treatment.

## Author Contributions

RJ performed the search for new publications and proceedings relevant to this study; reviewed the paper prior to submission. MI drafted the paper.

## Conflict of Interest Statement

RJ: Consulting/Advisory Boards for BMS, Merck, AstraZeneca, Pfizer, Roche as well as honoraria from each of them. MI declares no conflict of interest.
